# Revascularization and vital pulp therapy in immature molars with necrotic pulp and irreversible pulpitis: A case report with two‐year follow‐up

**DOI:** 10.1002/ccr3.2614

**Published:** 2019-12-19

**Authors:** Masoumeh Ramezani, Parisa Sanaei‐rad, Neda Hajihassani

**Affiliations:** ^1^ Department of Endodontics School of Dentistry Qazvin University of Medical Sciences Qazvin Iran

**Keywords:** immature tooth, regenerative endodontics, revascularization, vital pulp therapy

## Abstract

Management of teeth with inflamed pulp has been always a challenge. Revascularization and vital pulp therapy are suggested as procedures for successful treatment of immature molars diagnosed with pulp necrosis and irreversible pulpitis, respectively.

## Graphical abstract

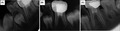

## 1 INTRODUCTION

Traditionally, the management of immature teeth with necrotic pulp or irreversible pulpitis has been always a challenge in endodontics. This is not encountered in adult patients and may be due to the wide‐open root apex and thin dentin walls of an immature tooth. The treatment options for such immature teeth are dependent on the condition of the pulp tissue. When the pulp is diagnosed as necrotic, the traditional treatment option for immature teeth is apexification with calcium hydroxide.^1^ The main disadvantage of this procedure is that the patient compliance is unavoidable due to the requirement of multiple visits and a long‐term period before treatment completion. The other procedure which lacks these limitations is the apexification with mineral trioxide aggregate as a barrier material, in the apical portion of the canal.^2^ Unfortunately, both of these methods suffer from the inability of the treated root to continue its natural development and may lead to thin fragile root walls. In the case of irreversible pulpitis, even after an external stimulus, such as cold or heat is removed, pain will be persistent.^3^ Herein, apexification is the standard treatment choice for irreversible pulpitis in teeth with open apex.^1^ Recently, regenerative endodontics (RE) has been introduced as an alternative for the treatment of teeth with irreversible pulpitis or necrotic pulp and open apex.^4^ One of the successful procedures of RE is the revascularization in which a bleeding is induced and the following blood clot provides a scaffold for migrated or resident stem cells to attach, proliferate, and differentiate into the vital components of pulp‐dentin complex.^5^ Vital pulp therapy is the treatment of choice for the immature teeth with reversible pulpitis and includes direct and indirect pulp capping and pulpotomy. However, this technique is not precluded if the irreversible pulpitis is diagnosed based on signs and symptoms along with clinical findings because these may not accurately reflect the pathologic conditions of the inflamed pulp.^6^

In this report, the immature mandibular right molar with necrotic pulp and left molar with pulpitis were treated using the new method of revascularization via induced bleeding and vital pulp therapy, respectively.

## 2 CASE REPORT

### 2.1 Revascularization in immature mandibular right first molar

**Figure 1:**
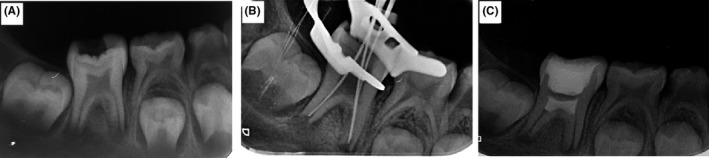
(A) Preoperative radiograph showing the right mandibular molar with periapical radiolucency, deep carious lesions, and PDL widening. (B) Working length determination and (C) postoperative final radiograph after induction of bleeding and MTA placement

An 8‐year‐old female with chief complaint of great pain in the right mandibular molar was referred to the school of dentistry, QUMS. She had this severe pain since one month ago. Although there were several teeth with decay, no dental treatment was performed until the day of examination. The medical history was noncontributory. In clinical evaluation, extraoral examination showed no facial asymmetry or swelling. Normal Palpation of the cervical and submandibular lymph nodes was also noticed. Intraoral examination showed no swelling or sinus tract but deep carious lesions were observed in the mandibular right first molar. In the diagnostic tests, the tooth showed mild response to percussion/palpation. However, it did not show any response to the different pulp tests. The probing depth was within normal limits (< 3 mm around the tooth), and no pathologic tooth mobility was observed. The tooth had an open apex in the radiographs, with periapical radiolucency, deep carious lesions and PDL widening (Figure 1A).

Taking clinical findings into account, the concluding diagnosis was pulp necrosis with symptomatic apical periodontitis. Due to the disadvantages of the traditional apexification method, the revascularization procedure was selected as our optimal treatment option and was performed according to the AAE Clinical Considerations for a Regenerative Procedure. The treatment procedure, risks, and benefits were completely explained to the patient, and then a written informed consent was obtained from the patient's legal guardians. At the first visit, the preparation of canals was performed. After local anesthesia using inferior alveolar nerve block (2% lidocaine with 1/80 000 Epinephrine; Daroupakhsh) and rubber dam isolation, then carries were removed by a round carbide bur and the access cavity was prepared using a fissure diamond bur and a high‐speed handpiece with water spray. The working length was determined by Root ZX electronic apex locator (J Morita MFQ) and confirmed with X‐ray (mesiobuccal: 15 mm; mesiolingual: 15 mm; distal: 14 mm) (Figure 1B).

The root canal system was slowly irrigated with 1.5% NaOCl (20 mL/canal for 5 minutes) and then saline, (20 mL/canal for 5 minutes). Throughout the procedure, irrigating needle was positioned at about 1 mm from root end. Later, the canals were dried using paper points and creamy Ca(OH)_2_ was placed by lentulo. The access cavity was then sealed with Zonalin (Kemdent), and the next visit was appointed for three weeks later. At the second visit, after clinical examination, it was demonstrated that the sensitivity to palpation/percussion had been resolved. Local anesthesia was performed via inferior alveolar nerve block with 3% mepivacaine (Daroupakhsh; without any vasoconstrictor). Then, the tooth was isolated with rubber dam, the temporary fillings were removed and the removal of intracanal medicament was performed by irrigating with 17% EDTA (30 mL/canal, for 5 minutes; Sigma‐Aldrich) and then final flush with saline (5 mL/canal, for 1 minute) Followed by drying with paper points. To induce bleeding, a precurved #25K‐file (Dentsply‐Maillefer) was rotated at 2 mm past the apical foramen. After blood clot formation, approximately 3 mm of MTA (Angelus) was placed over the canals and pulp chamber followed by the placement of wet cotton pellets on the top of the MTA. Then, the access cavity was sealed by the placement of the Cavit. At the third visit (the day after MTA placement), temporary fillings were removed to evaluate the setting of MTA and the access cavity was then sealed with zonalin (Figure 1C). Finally, the patient was referred to the department of pediatrics for the placement of stainless steel crown following coronal restoration with amalgam (gs‐80, SDI).

**Figure 2:**
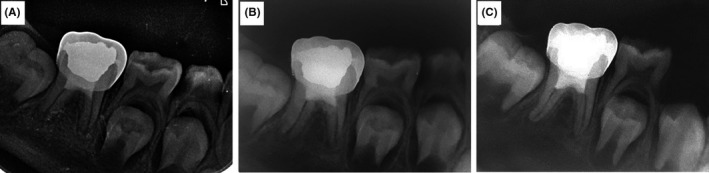
Follow‐up radiographs after revascularization. (A) 3‐mo, (B) 6‐mo, and (C) 12‐mo which show complete root development and healing of periapical lesion

In the three‐month follow‐up visit, the patient was asymptomatic and all signs and symptoms were removed (Figure 2A). Furthermore, the initiation of apical closure was obvious in six‐month follow‐up (Figure 2B). In the one‐year follow‐up, both mesial and distal roots showed root maturation and closing of the apical foramen (Figure 2C). The periapical lesion was also completely healed one year after revascularization.

### 2.2 Pulpotomy in immature mandibular left first molar

**Figure 3:**
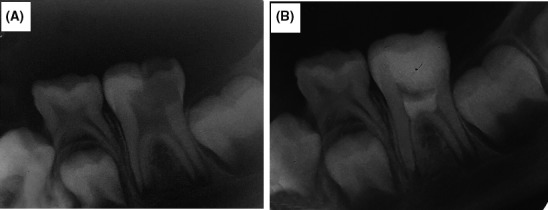
(A) preoperative radiograph showing the left mandibular molar with open apex and deep carious lesions, (B) postoperative radiograph after MTA placement canal orifices and the floor of pulp chamber

Four months after the revascularization in the right molar, the dental examination revealed that the mandibular left molar had deep carious lesions without any swelling or sinus tract. The patient's tooth has been sensitive to cold since one week ago. The tooth was also asymptomatic to percussion and palpation. The tooth did not respond to the pulp vitality tests such as heat and EPT. However, it showed response to cold test with sever lingering pain. The tooth had an open apex with deep carious lesions in the radiograph (Figure 3A). Based on clinical and radiographic examinations, the final diagnosis was symptomatic irreversible pulpitis without any periapical lesion. After local anesthesia using inferior alveolar nerve block (2% lidocaine with 1/80 000 Epinephrine) and rubber dam isolation, then carries were removed by a round carbide bur and the access cavity was prepared using a fissure diamond bur. Because of large and multiple pulpal exposure, full pulpotomy was then performed using another round carbide bur. Hemostasis was established by the application of cotton pellet moistened with 5.25% NaOCl for 5 minutes. Before MTA placement, the dentin was gently washed with saline and dried with cotton pellet to remove excess NaOCl. Then, MTA was directly placed in a 3‐mm layer over the canal orifices and the floor of pulp chamber followed by the placement of moist cotton pellet over its entire mass. The access cavity was temporarily sealed with Cavit (3M ESPE; Figure 3B), and the patient was re‐examined in the next day.

In the second visit, MTA setting was checked and the patient was referred to the department of pediatrics to restore the tooth with stainless steel crown following coronal restoration with amalgam (gs‐80, SDI).

**Figure 4:**
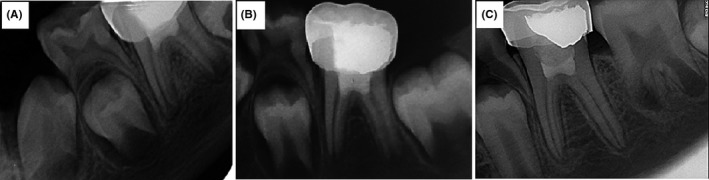
Follow‐up radiographs after pulpotomy. (A) 3‐mo follow‐up, (B) 9‐mo follow‐up, and (C) 24‐mo follow‐up which show complete root development and closed apex

In the three‐month follow‐up visit, the patient was asymptomatic and no signs and symptoms were observed. In nine‐month follow‐up, the periapical radiographs showed that the apices were almost closed with no sign of pathology. A long‐term follow‐up was performed for 24 months, and complete closure of apices was observed (Figure 4).

## 3 DISCUSSION

The treatment of pulpal necrosis or irreversible pulpitis in immature teeth with open apices has been a great challenge among dentists. Recently, according to the approach of regenerative endodontics, the technique of revascularization has been shown to be effective for the regeneration of pulp in immature teeth with pulp necrosis as well as induction of root formation.^5^, ^7^, ^8^ In the case of irreversible pulpitis, complete root development has been demonstrated in immature teeth by vital pulp therapy.^9^, ^10^ In the present case report, two immature teeth with necrotic pulp and irreversible pulpitis were treated by revascularization and pulpotomy, respectively, in an 8‐year‐old patient.

Revascularization needs three key steps for successful regeneration of the pulp tissue: disinfection of intracanal space, application of a scaffold to organize host stem cells, and an appropriate coronal seal. In the present report, in contrast to several previous case reports,^11^, ^12^, ^13^, ^14^ a low concentration of NaOCl (1.5%) was used to disinfect the canal and eliminate necrotic tissue so that a higher population of stem cells can be maintained viable in the microenvironment. Martin et al showed that 6% NaOCl significantly reduced the survival and odontogenic differentiation of apical papilla stem cells while 1.5% NaOCl increased the expression of DSPP in stem cells.^15^ After disinfection, it is recommended to use intracanal medicaments, either Ca(OH)_2_ or triple paste antibiotic (TAP). In this case, TAP was excluded due to its drawbacks such as tooth discoloration and relatively high expense.^16^ In addition, Ca(OH)_2_ has been demonstrated to have lower cytotoxicity to stem cells.^17^ In the revascularization procedure, the clinician uses the approach of tissue engineering for the regeneration of pulp tissue and maturation of root apex. Herein, a biodegradable scaffold serves as a temporary matrix for regenerating cells to attach, proliferate, and differentiate to mature and functional cells which produce pulp and dentin.^18^, ^19^ It is now well‐established that mesenchymal stem cells which migrate from different sources such as apical papilla, periodontal ligament, and bone marrow play a significant role in pulp and dentin regeneration.^20^ In the present case, migrating stem cells are suggested to be responsible for pulp regeneration, dentin production, and root development. The capability of mesenchymal stem cells for differentiation into odontoblast‐like cells has been demonstrated in several previous studies in vitro and in vivo.^21^, ^22^, ^23^

According to our clinical and radiographic examinations, mandibular left molar was diagnosed with irreversible pulpitis. Traditionally, it is regarded as an indication of root canal treatment (RCT).^24^ However, since an immature permanent tooth has thin root dentin walls, this treatment plan is challenging.^25^ In addition, there is increasing number of studies on vital pulp therapy of immature teeth with irreversible pulpitis that have demonstrated successful outcome.^9^, ^10^, ^26^ Therefore, we selected pulpotomy as a vital pulp therapy procedure to treat the immature left molar. In addition to the continuation of root development and closure of the apex, the other advantages of pulpotomy compared with apexification or RCT are rapid pain relief, patient compliance, and lower cost. As confirmed by the radiographs, MTA pulpotomy treatment allowed the immature root to complete its development and closure of the apex. The successful outcome of pulpotomy in the immature tooth with the diagnosis of irreversible pulpitis can be indicative of remaining vital pulp in the radicular portion. Taking these into account, it seems that the terminology of irreversible pulpitis which is currently based on the clinical signs and symptoms should be revised.

## CONFLICT OF INTEREST

The authors have no conflict of interest to declare.

## AUTHOR CONTRIBUTION

MR**:** reviewed the literature, developed the concept and design of the study, and performed the procedure. PS: reviewed the literature, involved in data analysis/interpretation, and drafted the manuscript. NH: involved in concept/design, analysis, and data collection.
